# Pickering Emulsion Stabilized by Chitosan-Modified *Saigae Tataricae Cornu* Particles for Improving the Oxidative Stability and In Vivo Pharmacokinetics of *Acorus tatarinowii* Schott Volatile Oil

**DOI:** 10.3390/ph19071027

**Published:** 2026-06-30

**Authors:** Xiaoxiao Lin, Zhichao Wang, Fei Luan, Xiaofei Zhang, Dongyan Guo, Bingtao Zhai, Liang Feng, Yajun Shi, Junbo Zou

**Affiliations:** 1 Shaanxi Province Key Laboratory of New Drugs and Chinese Medicine Foundation Research, School of Pharmacy, Shaanxi University of Chinese Medicine, No. 1, Shiji Avenue, Xi Xian New District, Xi’an 712046, China; 18391954985@163.com (X.L.); m18049365980@163.com (Z.W.); 2051145@sntcm.edu.cn (F.L.); 2051028@sntcm.edu.cn (X.Z.); winter180@163.com (D.G.); 2051136@sntcm.edu.cn (B.Z.); 2 Innovation Center for Industry-Education Integration of Pediatrics and Traditional Chinese Medicine, State Key Laboratory of Natural Medicines, School of Traditional Chinese Pharmacy, China Pharmaceutical University, Nanjing 211198, China; wenmoxiushi@163.com; 3 Jiangning Hospital of Chinese Medicine Affiliated to China Pharmaceutical University, Nanjing 211198, China; 4 Nanjing Jiangning District Hospital of Chinese Medicine, Nanjing 211198, China

**Keywords:** *Acorus tatarinowii* Schott volatile oil, Pickering emulsion, *Saigae Tataricae Cornu*, stability, bioavailability

## Abstract

**Background/Objectives**: *Acorus tatarinowii* Schott volatile oil (ATVO), a bioactive component of traditional Chinese medicine, is susceptible to light-induced oxidation and compositional changes. This study aimed to develop a chitosan-modified *Saigae Tataricae Cornu* particle (MSTC)-stabilized Pickering emulsion (PE) to improve the light-oxidative stability and in vivo disposition of ATVO. **Methods**: *Saigae Tataricae Cornu* particles were modified with chitosan and used to prepare an oil-in-water PE encapsulating ATVO. Particle wettability, morphology, structural interactions, emulsion type, interfacial distribution, droplet size, and zeta potential were characterized. The light-oxidative stability of ATVO was evaluated under light using peroxide value, malondialdehyde content, and gas chromatography-mass spectrometry (GC-MS) analysis. The pharmacokinetic behavior of α-asarone and β-asarone was further investigated in rats. **Results**: Chitosan modification increased the contact angle of *Saigae Tataricae Cornu* particles from 65.37° to 83.23°, indicating improved wettability and interfacial affinity. The resulting PE showed good physical stability, with a droplet size of 2.51 μm and a zeta potential of +32.00 mV. Confocal laser scanning microscopy (CLSM) confirmed that MSTC particles adsorbed at the oil–water interface and encapsulated ATVO within the oil droplets. Compared with free ATVO and the physical mixture, the PE reduced peroxide and malondialdehyde formation, slowed light-induced changes in volatile components, and better preserved major bioactive constituents. Pharmacokinetic analysis showed that the plasma concentration-time curve from 0 to t (AUC_0–t_) and maximum plasma concentration (C_max_) of α-asarone increased by 2.02- and 2.47-fold, respectively, whereas the effect on β-asarone was relatively limited. **Conclusions**: MSTC-stabilized PE provides an effective interfacial-barrier strategy for protecting ATVO against light-oxidative deterioration. This study highlights the potential of modified natural medicinal particles as green stabilizers for improving the stability, quality consistency, and delivery performance of volatile-oil-containing traditional Chinese medicine preparations.

## 1. Introduction

Volatile oils are widely recognized as important chemical constituents responsible for the pharmacological effects of aromatic traditional Chinese medicines (TCM). They are involved in various biological activities, including antibacterial, anti-inflammatory, immunomodulatory, and neuropsychological regulatory effects, and have important application value in pharmaceutical formulations, natural product development, food and daily chemical products [1,2,3,4]. However, volatile oil components usually possess high volatility and lipophilicity, and their molecular structures often contain unsaturated bonds and oxidation-sensitive functional groups, such as aldehyde and ketone groups. When exposed to oxygen, light, or heat, volatile oils are prone to oxidative transformation and volatilization loss, accompanied by the formation of oxidation products such as peroxides and malondialdehyde [5,6]. Therefore, improving the stability of volatile oils remains an important prerequisite for ensuring the reliability of clinical efficacy and further realizing the industrial standardization of related formulations.

At present, various formulation technologies have been used to reduce the deterioration of volatile oils, including microencapsulation, cyclodextrin inclusion, and liposomal encapsulation [7,8,9,10]. Although these approaches can provide a certain degree of protection, their practical applications are often limited by complex preparation processes, relatively high production costs, limited long-term stability, and, in some cases, the need for additional excipients. These limitations may reduce the simplicity and translational feasibility of the formulation system [11,12]. Therefore, it remains necessary to develop alternative strategies that can provide durable protection while considering manufacturing practicality and minimizing the use of non-native additives.

In recent years, Pickering emulsification has emerged as a promising platform for encapsulating unstable lipophilic substances [13,14,15]. In PE systems, solid particles replace conventional surfactants and are immobilized at the oil–water interface, where they form a compact interfacial particle layer to suppress droplet coalescence and phase separation [16,17]. This stabilization mode offers several advantages for formulation design, including high physical stability, the potential to reduce interfacial-mediated oxidation, and the possibility of using biocompatible particulate materials [16,18,19,20,21]. However, the stability and performance of PE largely depend on the properties of the particle stabilizers. Effective interfacial adsorption requires particles with appropriately balanced wettability, suitable surface charge, and sufficient interfacial anchoring strength [22,23,24]. Many naturally derived powders are highly hydrophilic and exhibit weak adsorption at the oil–water interface, which limits their ability to stabilize emulsions and restricts the controllability and application scope of PE carrier systems [24,25,26].

Particle design technology provides a feasible approach to overcome these limitations. By regulating particle surface structure, wettability, and interfacial affinity through physical, chemical, or composite modification methods, the adsorption ability of particles at the oil–water interface can be enhanced, thereby improving the stability and controllability of Pickering emulsion systems [27]. Among natural polymer materials, chitosan is a biocompatible cationic polysaccharide derivative derived from natural chitin, with favorable biodegradability, film-forming ability, and mucoadhesive properties. It has been widely used in drug delivery, biomedical materials, and functional formulation research [28]. The abundant amino and hydroxyl groups in its molecular structure can interact with proteins, polysaccharides, and other natural polymers through hydrogen bonding and electrostatic interactions, thereby regulating the surface charge, wettability, and interfacial behavior of composite particles [28,29,30]. Therefore, chitosan can serve as a suitable surface modification material to improve the interfacial adsorption properties of natural medicinal powders and provide a material basis for constructing biocompatible Pickering emulsion systems [31,32].

Lingzhu Powder is a representative classic TCM preparation containing volatile oil. Its prescription consists of animal-derived materials, including STC and artificial bezoar; mineral-derived material, cinnabar; resin-derived material, amber; as well as Bombyx Batryticatus, Arisaema cum Bile, and ATVO. Clinically, it is commonly used for clearing heat, resolving phlegm, calming convulsions, and relieving spasms [33]. ATVO contains various bioactive constituents, including methyleugenol, α-asarone, and β-asarone, which are associated with anti-inflammatory activity, lipid metabolism regulation, and potential cardiovascular protection [3,34,35]. However, ATVO is particularly susceptible to quality deterioration under thermal or oxidative conditions, resulting in the loss of active constituents and reduced formulation reliability. To address these issues, our research group previously combined particle design technology with Pickering emulsification strategies and successfully constructed corresponding PE systems using prescription-derived particles from Lingzhu Powder. Specifically, previous studies modified cinnabar, a mineral inorganic powder, using a solvent evaporation strategy, and regulated the surface properties of artificial bezoar- and amber-related systems using melting or PEG-related modification strategies [33,36,37]. These studies mainly evaluated the stability of ATVO under thermal conditions or simulated oxidative environments induced by metal ions or ozone, and demonstrated the feasibility of using prescription-derived particles as PE stabilizers after particle design. However, previous studies mainly focused on mineral, resin-derived, or complex prescription particles, whereas the particle design and interfacial functionalization of protein-rich animal-derived medicinal powders remain insufficiently explored.

On this basis, the present study further focused on STC powder, an animal-derived medicinal powder rich in proteinaceous and fibrous components. STC has good biocompatibility and a long history of medicinal use, but its native powder is highly hydrophilic and exhibits limited adsorption capacity at the oil–water interface, making it difficult to directly serve as an efficient PE stabilizer [26]. Through possible hydrogen bonding and electrostatic interactions between the protein matrix of the animal-derived medicinal powder and chitosan polysaccharide chains, the wettability and interfacial adsorption ability of the particles can be improved, thereby transforming them into functional interfacial particles suitable for stabilizing PE. In addition, this study further extended the stability evaluation scenario from the thermal, metal ion-, and ozone-induced oxidative models used in previous studies to light-induced, light-oxidative degradation. Continuous light was used as an accelerated light-oxidative stress condition to evaluate the protective effect of the MSTC-stabilized PE on ATVO.

In summary, guided by the “particle design–Pickering emulsion” bridging strategy, this study aimed to modify STC powder with chitosan to regulate its wettability and enhance its interfacial anchoring ability. This strategy was designed to construct a PE carrier without introducing additional conventional surfactants, thereby achieving efficient encapsulation of ATVO and improving its oxidative stability. Furthermore, the physicochemical properties and interfacial behavior of the system were systematically characterized, and light-induced accelerated oxidation evaluation and comparative in vivo pharmacokinetic studies were performed to elucidate the protective and delivery mechanisms of this PE system under environmental stress and physiological conditions. This study provides a green and translatable stabilization strategy for volatile-oil-containing traditional Chinese medicine solid preparations.

## 2. Results

### 2.1. Physicochemical Characterization of MSTC

**Figure 1:**
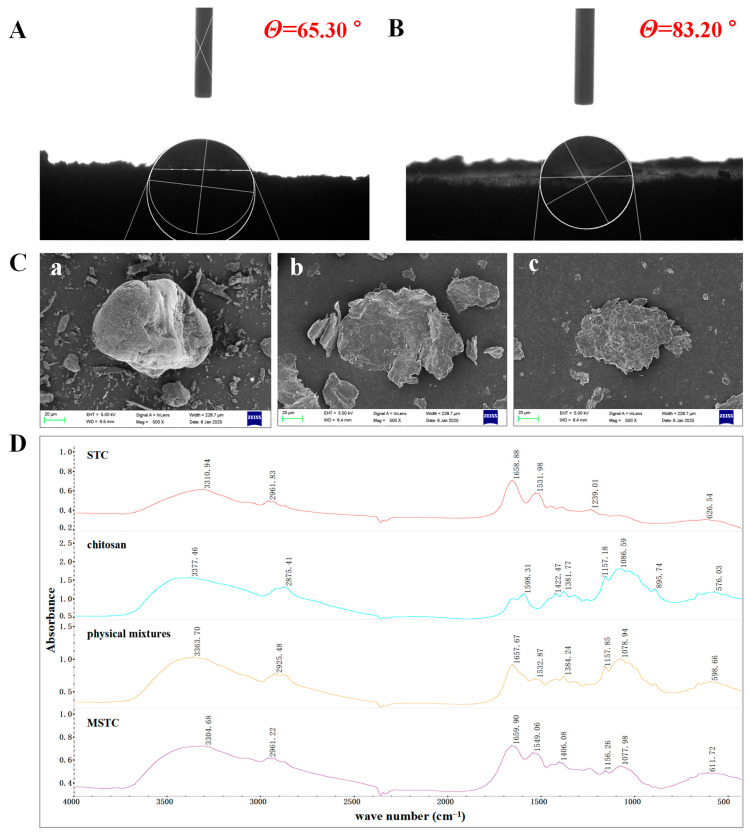
*Saigae Tataricae Cornu* particles water phase contact angle diagram (**A**); MSTC water phase contact angle diagram (**B**); SEM of *Saigae Tataricae Cornu* (**a**), chitosan (**b**) and MSTC (**c**) (**C**); infrared spectra of *Saigae Tataricae Cornu* particles, chitosan, physical mixtures and MSTC (**D**).

As shown in Figure 1A,B, the contact angle of the original STC powder was 65.37 ± 1.30°, whereas that of MSTC increased to 83.23 ± 2.15° after chitosan modification, indicating an obvious change in particle wettability (*n* = 3, *p* < 0.001).

The SEM and EDS analyses revealed morphological and elemental differences among STC, chitosan, and MSTC (Figure 1C; Table 1). The raw STC powder showed irregular block-like structures with relatively small particle sizes and was mainly composed of C, N and O with minor amounts of P, S, Ca and Mg. Chitosan appeared as smooth flakes and was mainly composed of C, O and N. After modification, MSTC exhibited larger serrated lamellar structures. Its main elements were C (74.71%) and O (15.45%), while the contents of P and S decreased and N was not detected.

As shown in Figure 1D, STC exhibited characteristic amide I and amide II bands at 1657 cm^−1^ and 1519 cm^−1^, respectively, as well as peaks at 2961 cm^−1^ and 614 cm^−1^. Chitosan showed broad absorption around 3300 cm^−1^, bands at 2900–2850 cm^−1^, and a characteristic peak at 1079 cm^−1^. The physical mixture showed the spectral features of both components. In the FTIR spectrum of MSTC, the amide I and amide II bands were weakened or disappeared, while the broad absorption band around 3300 cm^−1^ was enhanced and slightly red-shifted. The characteristic peaks of STC and chitosan were still observed, indicating the coexistence of both components in MSTC.

**Figure 2:**
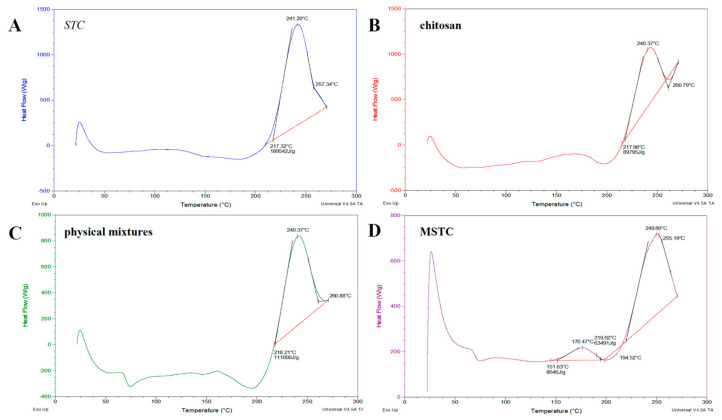
DSC curves of *Saigae Tataricae Cornu* particles (**A**), chitosan (**B**), physical mixtures (**C**), and MSTC (**D**).

DSC thermograms are shown in Figure 2. STC exhibited a sharp endothermic peak at 257.34 °C and a weaker thermal transition at 217.32 °C. Chitosan showed a prominent thermal transition at 260.79 °C and a minor shoulder peak at 217.73 °C. The physical mixture mainly showed a superposition of the thermal transitions of the two components, with a major endothermic peak at 260.88 °C and a secondary peak at 218.21 °C. In contrast, MSTC exhibited new endothermic peaks at 178.47 °C and 194.92 °C, while the original high-temperature peaks at 248.80 °C and 205.19 °C were markedly weakened.

Taken together, the above characterization results demonstrate the successful chitosan modification of STC particles. SEM/EDS confirmed surface morphological reconstruction and elemental redistribution after modification. FTIR revealed possible hydrogen bonding and electrostatic interactions between the protein matrix of STC and chitosan, while DSC further showed changes in the thermal behavior of the modified particles. In addition, the increased contact angle indicated that chitosan modification altered particle wettability and enhanced oil–water interfacial affinity. These changes collectively suggest that MSTC possessed improved interfacial properties suitable for stabilizing PE.

### 2.2. Characterization of PE

**Figure 3:**
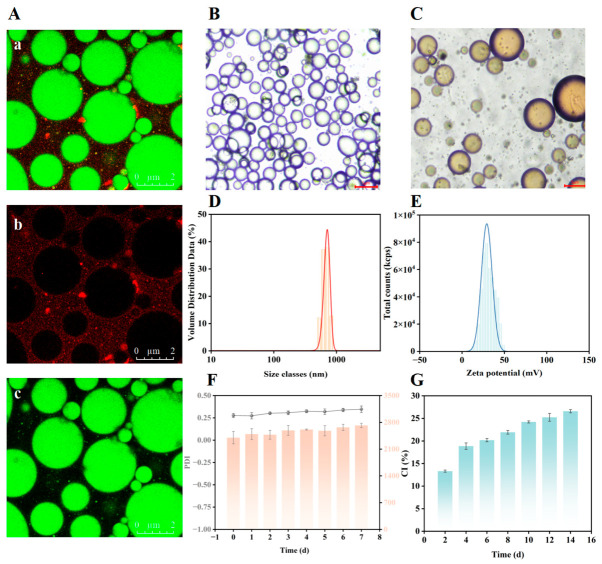
Characterization of MSTC-stabilized PE. CLSM images of PE (**A**); Nile Red-labeled oil phase (**a**), Nile Blue-labeled MSTC particles (**b**), and merged image (**c**); methylene blue staining image (**B**); Sudan III staining image (**C**); droplet size distribution of PE (**D**); zeta potential of PE (**E**); changes in droplet size and PDI during 7 days of storage at room temperature (**F**); and CI changes during storage (**G**).

Microscopic observation showed that methylene blue, a water-soluble dye, was distributed in the continuous external phase, whereas Sudan III, an oil-soluble dye, stained the dispersed oil droplets (Figure 3B,C). This confirmed that the MSTC-stabilized PE was of the oil-in-water (O/W) type, with ATVO dispersed as the internal oil phase.

As shown in Figure 3A, the green fluorescence from the labeled oil phase was mainly confined inside the droplets, while the red fluorescence from Nile Blue-labeled MSTC was distributed around the droplet interfaces. The merged image showed that the green oil cores were surrounded by red fluorescent shells, indicating that MSTC was adsorbed at the oil–water interface and formed a stable interfacial particle layer.

As shown in Figure 3D, the PE stabilized by MSTC exhibited a relatively uniform droplet size distribution, with an average droplet diameter of 2510 ± 0.75 nm and a polydispersity index (PDI) of 0.2879 ± 0.23. In addition, the zeta potential of the PE stabilized by MSTC was +32.00 ± 1.37mV (Figure 3E), indicating that the prepared PE had good electrostatic stability.

The storage stability of the PE was further evaluated by monitoring the changes in droplet size, PDI, and creaming index (CI). As shown in Figure 3F, the droplet size and PDI showed only slight increases during 7 days of storage, suggesting that no obvious droplet aggregation occurred during this period. As shown in Figure 3G, the CI value gradually increased with storage time, indicating slight creaming of the emulsion. However, the CI value remained below 30% during the observation period, suggesting that no severe phase separation occurred.

### 2.3. Effects of PE on the Accelerated Oxidative Stability of ATVO Under Light

#### 2.3.1. Volatile Oil Retention Rate

The retention rate of volatile oil in each group under light exposure was calculated to reflect the degree of volatile oil loss. After storage in a light chamber at 4500 lx ± 500 lx for 1, 3, and 5 days, the light-exposure retention rates of the ATVO group were 89.14 ± 0.61%, 75.74 ± 0.65%, and 64.38 ± 0.30%, respectively. The retention rates of the physical mixture group were 91.75 ± 0.53%, 79.60 ± 0.39%, and 67.75 ± 0.62%, respectively. The retention rates of the PE group were 93.53 ± 0.29%, 81.85 ± 0.37%, and 71.56 ± 0.48%, respectively. The experimental results are shown in Table 2. Compared with the ATVO group, the physical mixture group showed an increased volatile oil retention rate, while the PE group exhibited a more pronounced retention effect.

#### 2.3.2. Peroxide Contents

After 1, 3, and 5 days of light exposure, the PE group exhibited significantly lower peroxide content than the ATVO group (*p* < 0.001), as shown in Table 3. These results indicate that the PE group had the lowest peroxide value and the lowest oxidation level. This suggests that the emulsion effectively inhibited peroxide formation and improved the oxidative stability of ATVO.

#### 2.3.3. Malondialdehyde (MDA) Contents

The standard curve equation for MDA was *Y* = 0.867*x* + 0.0025, with R^2^ = 0.9988. As shown in Table 4, the MDA content in the PE was significantly lower than that in the untreated group after 1, 3, and 5 days of light exposure (*p* < 0.01). These results indicate that the PE provided stronger protection against light-induced oxidation than free ATVO. The physical mixture also reduced MDA formation after 3 and 5 days of light exposure (*p* < 0.01), but the PE group showed the lowest MDA level overall.

### 2.4. GC-MS Analysis of Volatile Component Composition of ATVO

**Figure 4:**
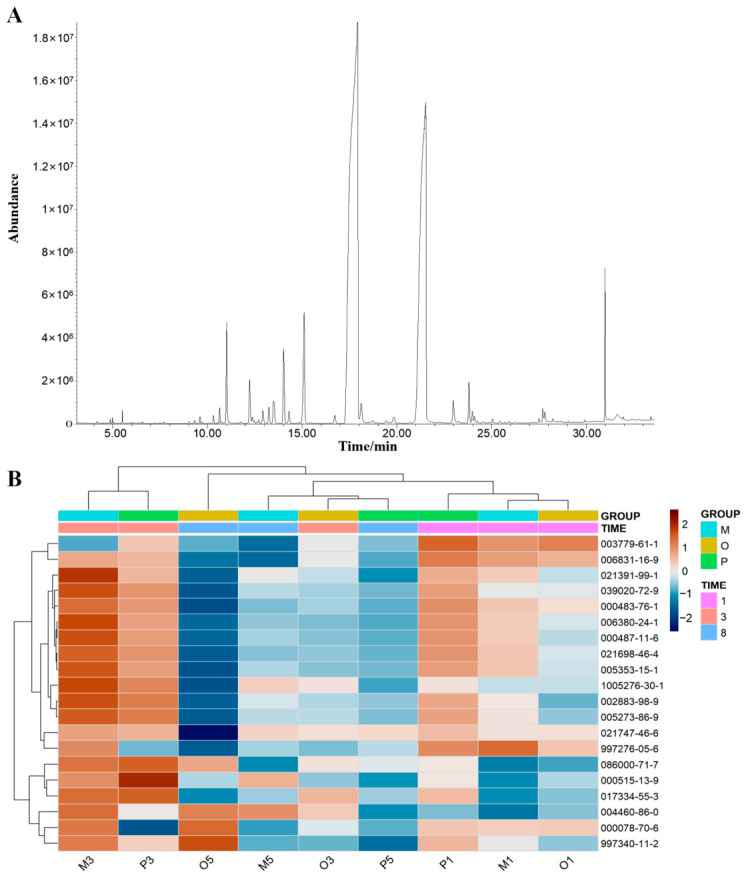
GC-MS total ion current diagram of ATVO (**A**); hierarchical clustering heatmap (**B**); M: physical mixture group; O: ATVO group; P: PE group.

By referencing the NIST 14.L database, the GC-MS total ion chromatogram (TIC) of ATVO was analyzed (Figure 4A). The chromatographic profile exhibited good peak resolution, symmetrical peak shapes, no obvious overlap between adjacent peaks, and a smooth baseline, indicating that this method was suitable for the analysis of volatile components in ATVO. For each retained compound, the database output, including retention time (RT), library-assigned compound name (Library/ID), CAS number, and match quality score (Qual), is provided in Supplementary Table S5.

The GC-MS results showed that ATVO contained multiple volatile components. The major components included α-asarone, β-asarone, γ-asarone, and methyleugenol. In addition, several minor components with relatively low contents were also detected, such as shyobunone, isocalamendiol, 2,4,5-trimethoxybenzaldehyde, and other terpenoid or oxygen-containing small-molecule compounds. These components together constitute the chemical composition basis of ATVO and provide an important foundation for the subsequent evaluation of component changes during light-induced oxidation and the protective effect of PE.

Hierarchical clustering was performed using the top 20% volatile compounds, ranked by relative abundance (Figure 4B). The results showed distinct grouping patterns among the different samples. Specifically, P3 clustered closely with M3, P1 with M5, O3 with P5, and M1 with O1, suggesting that different formulation systems and light-exposure durations could affect the relative contents of volatile components in ATVO. With the prolongation of light-exposure time, some volatile components in the O group decreased markedly, whereas the P group still maintained relatively higher levels of volatile components after 1, 3, and 5 days of light exposure. These results indicate that PE could delay the changes in both major and minor volatile components of ATVO.

### 2.5. Analysis of Volatile Component Content Changes

#### 2.5.1. Screening of Differential Components

**Figure 5:**
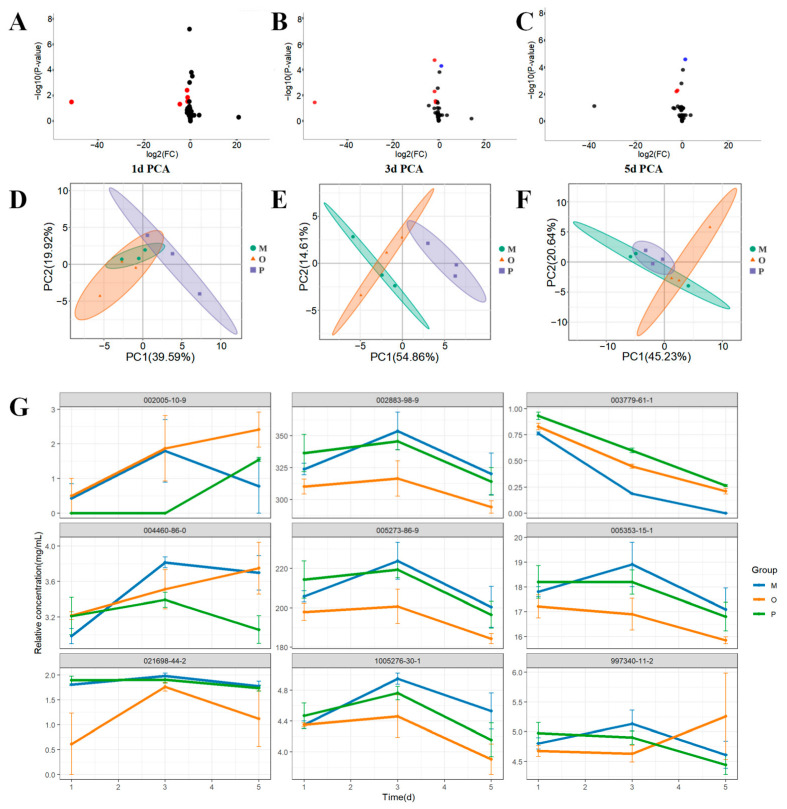
Screening of differential components, relative content changes, and PCA analysis under light exposure. Volcano plots of differential components after 1 d (**A**), 3 d (**B**), and 5 d (**C**) of light exposure; Blue dots represent upregulated components, black dots represent components with no significant change, and red dots represent downregulated components. PCA score plots of different groups after 1 d (**D**), 3 d (**E**), and 5 d (**F**) of light exposure. Relative content changes of differential components in different groups (**G**). O, free ATVO group; M, physical mixture group; P, MSTC-stabilized PE group.

As shown in Figure 5A–C, untreated ATVO was used as the blank control, and the volatile components of ATVO exposed to light for 1, 3, and 5 days were compared with those of untreated ATVO to screen differential volatile components induced by light. The results revealed 5, 6, and 3 differential compounds at each respective time point. After removing duplicates, a total of nine differential volatile compounds were identified. These compounds were 6H-Dibenzol\[b,d]pyran-6-one (CAS No.: 002005-10-9), (E)-3,7-dimethylocta-1,3,6-triene (CAS No.: 003779-61-1), α-Asarone (CAS No.: 002883-98-9), β-Asarone (CAS No.: 005273-86-9), 2,4,5-Trimethoxybenzaldehyde (CAS No.: 004460-86-0), γ-Asarone (CAS No.: 005353-15-1), 4a(2H)-Naphthalenol (CAS No.: 021698-44-2), shyobunone (CAS No.: 005353-15-1), 1,5,6,7,8,8a-hexahydro-1-methyl-6-methylene-4-(1-methylethyl)- (CAS No.: 1005276-30-1), and isocalamendiol (CAS No.: 997340-11-2).

#### 2.5.2. Changes in the Content of Differential Components

As shown in Figure 5G, the relative contents of the screened differential components were further compared among the O, M, and P groups. With the prolongation of light-exposure time, these components in the O group exhibited either a decreasing or increasing trend. However, these components showed more stable variation trends in the PE group and physical mixture group, although the fluctuations in the physical mixture group were more pronounced. Notably, the content of compound 003779-61-1 decreased to zero after 5 d of light exposure, whereas it was well retained in the PE group. In addition, for the major components of ATVO, such as α-asarone (002883-98-9), β-asarone (005273-86-9), and γ-asarone (005353-15-1), the PE effectively delayed the changes in their relative contents, indicating that PE exerted a protective effect on the volatile components in ATVO.

#### 2.5.3. Discrimination of Differential Compounds by Principal Component Analysis (PCA)

As shown in Figure 5D–F, PCA was performed to evaluate the differences and relationships in volatile component profiles among the three treatment groups (M, O, and P) after light exposure for 1, 3, and 5 days. In the PCA score plots, a shorter distance between sample points indicates a more similar compositional profile, whereas a greater distance indicates more pronounced differences among treatment systems.

After 1 day of light exposure, PC1 and PC2 explained 39.59% and 19.92% of the total variance, respectively. The M, O and P groups were relatively close to each other, indicating that the compositional differences among the three formulation systems were not yet obvious after short-term light. After 3 days of light exposure, PC1 and PC2 explained 54.86% and 14.61% of the total variance, respectively. Compared with day 1, the separation among the M, O, and P groups became more evident, with no obvious overlap. This suggests that the influence of different treatment systems on ATVO volatile components increased with prolonged light. The clear separation between the O and P groups indicates distinct differences between free ATVO and PE-encapsulated ATVO, while the M group showed intermediate characteristics, suggesting a limited protective effect of the physical mixture. After 5 days of light exposure, PC1 and PC2 accounted for 45.23% and 20.64% of the total variance, respectively. Partial proximity or overlap was observed among the groups, indicating that all the samples underwent certain compositional changes after prolonged light. However, compared with the O group, the P group showed smaller overall variations, suggesting that the PE helped maintain the relative stability of ATVO volatile components.

Overall, the PCA results indicate that the differences in volatile component composition among the treatment systems changed with the duration of light exposure. The O group was more susceptible to light-induced compositional changes, the M group showed intermediate behavior, and the P group exhibited relatively smaller fluctuations. These findings are consistent with the differential component analysis and further support the protective effect of the MSTC-stabilized PE on ATVO.

### 2.6. Analysis of Volatile Component Composition Changes

**Figure 6:**
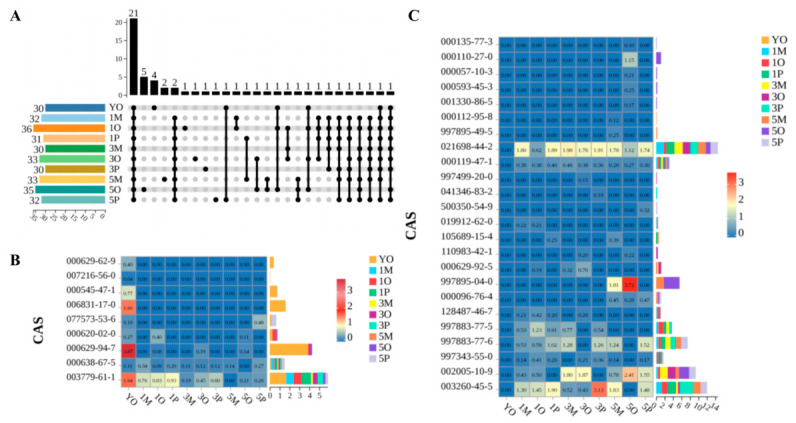
Qualitative analysis of volatile components under light-exposure conditions. Screening of qualitative components among different groups (**A**); heatmap of disappeared components (**B**); and heatmap of newly generated components (**C**).

Using untreated ATVO as the blank control, light-exposure conditions were found to affect not only the content changes of volatile oil components but also their composition, as reflected by the generation or disappearance of certain components. As shown in Figure 6A, with the extension of light-exposure time, the total number of volatile components in the O group significantly increased, particularly in the 1O group, which reached 36 components. This indicates that light exposure promoted the oxidation and generation of certain components in the volatile oil. In contrast, the P group exhibited smaller changes in component numbers across different time points and maintained a higher degree of overlap with the YO group, indicating that the PE system offers protective effects on the composition of volatile oil components.

Regarding newly generated components, as shown in Figure 6B, multiple new volatile components were produced in the O and M groups after light exposure compared with untreated ATVO, including components with CAS numbers 000110-27-0, 000057-10-3, 000593-45-3, and 997895-49-5. These components were either absent or present at very low concentrations in the P group, suggesting that the PE system effectively inhibits the formation of oxidation products. In terms of disappearing components, as shown in Figure 6C, the components 000629-62-9, 007216-56-0, 000545-47-1, and 006831-17-0 disappeared in the O, M, and P groups compared to crude oil. However, in the P group, the levels of key components such as 077573-53-6, 000638-67-5, and 003779-61-1 remained relatively stable, demonstrating that the PE system helps reduce the loss of certain volatile components.

Overall, the PE preserved the original volatile components more effectively than the O and M groups. It also delayed oxidation, indicating improved light stability of ATVO.

### 2.7. Physicochemical Property Analysis of Quantitative and Qualitative Components

The density, boiling point, molecular weight, flash point, LogP value, and refractive index of newly generated, disappeared, and quantitatively changed components were systematically analyzed to compare the physicochemical characteristics of different volatile oil component categories.

**Figure 7:**
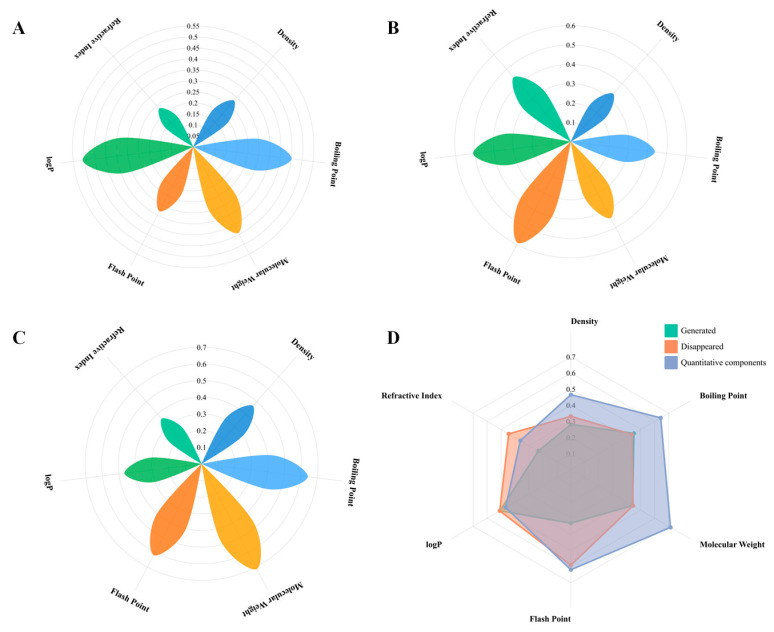
Petal diagram of physicochemical properties for generated (**A**), disappeared (**B**), and quantitative components (**C**); radar chart of physicochemical property distribution for both generated, disappeared, and quantitative components (**D**).

The petal plots showed that the newly generated components (Figure 7A) exhibited a relatively concentrated distribution of physicochemical parameters, with comparatively higher LogP values, moderate molecular weight and boiling point, and relatively lower density and refractive index. As shown in Figure 7B, the disappeared components were characterized by a prominently elevated flash point, accompanied by higher LogP values and refractive indices, indicating stronger hydrophobicity and enhanced thermal-related properties. The quantitatively changed components (Figure 7C) displayed the highest molecular weight and boiling point, with a relatively high flash point, while the remaining parameters were more evenly distributed. The integrated radar plot (Figure 7D) further demonstrated that quantitatively changed components contributed most prominently in terms of molecular weight, boiling point, and density; disappeared components were more pronounced in flash point and LogP; whereas newly generated components generally remained at moderate or relatively lower levels across most parameters. Overall, distinct differences in molecular scale, thermal properties, and hydrophobicity were observed among the three categories, which may underlie the mechanisms responsible for their generation, disappearance, and quantitative variation.

### 2.8. Analysis of Pharmacokinetic Characteristics

**Figure 8:**
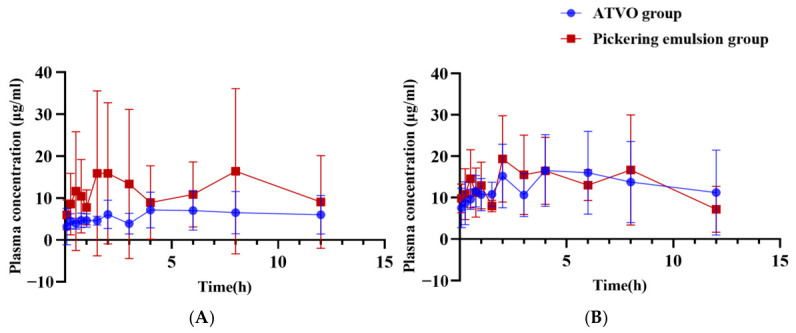
Mean plasma concentration-time curves of α-asarone (**A**) and β-asarone (**B**) in rats.

As shown in Figure 8 and Table 5, the pharmacokinetic parameters and plasma concentration-time profiles showed that the PE affected the two asarone isomers differently. AUC_0–t_ and AUC_0–∞_ reflect the overall systemic exposure of the drug in vivo, C_max_ represents the maximum plasma concentration, T_max_ represents the time to reach the maximum plasma concentration, and T_1/2_ and MRT are associated with the elimination and residence characteristics of the drug in vivo.

For α-asarone, compared with the ATVO group, the AUC_0–t_ of the PE group increased from 72.47 ± 33.89 mg/L·h to 146.67 ± 128.07 mg/L·h, representing an approximately 2.02-fold increase. The AUC_0–∞_ increased from 149.99 ± 101.70 mg/L·h to 594.26 ± 912.79 mg/L·h, representing an approximately 3.96-fold increase. The C_max_ increased from 9.91 ± 4.90 mg/L to 24.51 ± 21.13 mg/L, representing an approximately 2.47-fold increase. Meanwhile, T_max_ was shortened, while T_1/2_ and MRT_0–∞_ showed an increasing trend. These results suggest that the PE may increase the in vivo exposure of α-asarone and influence its residence characteristics. The plasma concentration-time profiles also showed that the PE group tended to exhibit a more rapid increase in plasma concentration during the early phase and maintained relatively higher plasma concentrations at multiple time points, further suggesting a potential increase in the overall exposure of α-asarone. For β-asarone, the AUC_0–t_ value in the PE group was close to that in the ATVO group, whereas AUC_0–∞_ and C_max_ showed only slight numerical increases. In contrast, T_1/2_ and MRT_0–∞_ showed numerical decreasing trends, suggesting that the PE may have a limited effect on the overall exposure and disposition characteristics of β-asarone.

Overall, the MSTC-stabilized PE showed different numerical trends in the pharmacokinetic parameters of the two asarone isomers. The numerical increase in systemic exposure was more apparent for α-asarone, whereas the effect of the PE on β-asarone was relatively limited.

## 3. Discussion

### 3.1. Chitosan Modification and Interfacial Functionalization of STC Particles

This study aimed to address the oxidative deterioration, volatilization loss, and compositional changes of ATVO under light exposure by constructing a PE system stabilized with MSTC. STC powder is rich in proteinaceous and fibrous components and has certain biocompatibility and prescription-derived advantages. However, the native particles are highly hydrophilic and exhibit limited adsorption capacity at the oil–water interface, making them unsuitable as efficient PE stabilizers. This is consistent with previous findings that natural protein-based powders are often restricted in emulsion stabilization due to wettability-related limitations [26,38]. Therefore, regulating the surface properties of STC particles through chitosan modification is critical for endowing them with interfacial stabilization capability [28,30,31].

Under weakly acidic conditions, the amino groups of chitosan can be protonated to form positively charged -NH^3+^ groups, which may interact with amide, hydroxyl, carboxyl, and other functional groups on the surface of STC particles through hydrogen bonding, electrostatic interactions, and intermolecular entanglement. The SEM/EDS, FTIR, and DSC results collectively indicate that the modification process was not a simple physical mixture, but involved surface morphological reconstruction, elemental redistribution, and protein-polysaccharide interactions. This composite modification regulated the hydrophilic–lipophilic balance of the particles and made MSTC more favorable for adsorption at the oil–water interface. Previous studies have shown that when particle wettability approaches an intermediate range, the interfacial adsorption energy is relatively high and particles are less likely to detach from the oil–water interface, thereby facilitating the formation of stable PE [13,19,26,35]. Therefore, chitosan modification effectively improved the interfacial functionalization of STC particles and provided a material basis for subsequent emulsion stabilization.

### 3.2. Stabilization Mechanism and Physical Stability of MSTC-Stabilized PE

The formation of the MSTC-stabilized PE was not governed by a single factor, but resulted from the combined effects of suitable wettability, interfacial adsorption, particle-film formation, steric hindrance, and electrostatic repulsion. CLSM results showed that MSTC particles were enriched on the surface of ATVO oil droplets and formed a continuous interfacial particle layer. This particle layer could reduce the oil–water interfacial free energy and form a physical barrier between adjacent droplets, thereby inhibiting droplet coalescence, flocculation and phase separation. This interfacial-barrier-mediated stabilization mechanism is consistent with previous reports that PE interfacial films can retard lipid oxidation through a blocking effect [14,39]. In addition, droplet size distribution, PDI, and zeta potential further reflected the physical stability basis of this system. A relatively uniform droplet distribution may reduce the risk of sedimentation or creaming caused by droplet size differences. Meanwhile, the relatively high positive charge on the droplet surface may be derived from the protonated amino groups of chitosan, which can enhance electrostatic repulsion between droplets and reduce aggregation tendency [28,30,31]. Therefore, the stability of the MSTC-stabilized PE was mainly attributed to the steric hindrance provided by the interfacial particle layer and the electrostatic repulsion generated by surface charges, which together maintained the dispersion stability of the emulsion system [13,40,41].

### 3.3. Light-Oxidative Protection of ATVO and Possible Oxidation Pathways

ATVO contains various volatile and oxidation-sensitive constituents, such as α-asarone, β-asarone, γ-asarone, and methyleugenol. These compounds contain unsaturated side chains, methoxy-substituted aromatic structures or other oxidation-labile moieties, and are therefore prone to volatilization loss, oxidative degradation, isomerization or further transformation under light and oxygen exposure. Thus, the quality changes of ATVO under light are reflected not only by the decrease in active constituents but also by the generation of new components and the disappearance of original components. In this study, the PE group showed better volatile oil retention and reduced peroxide value and MDA levels, indicating that it inhibited both primary and secondary oxidation of ATVO to some extent. Peroxide value mainly reflects the formation of peroxides and hydroperoxides in the early stage of oxidation, whereas MDA reflects secondary oxidation products generated from the further decomposition of unsaturated components [5,42]. Therefore, the reduction in both indicators suggests that the MSTC-stabilized PE could delay the light-induced oxidation of ATVO. Regarding the possible oxidation pathway, unsaturated volatile components in ATVO may first form radical intermediates under light exposure, which subsequently react with oxygen to generate peroxyl radicals and hydroperoxides. These intermediates can further decompose into aldehydes, ketones, alcohols, or other small-molecule oxidation products. Asarone-type constituents contain methoxy-substituted aromatic rings and unsaturated side chains, and may undergo double-bond isomerization, side-chain oxidation or oxidative cleavage [43,44]. The newly generated components observed by GC-MS may be regarded as chemical markers of light-induced oxidation and compositional drift, although their specific pharmacological and toxicological significance requires further investigation. The lower abundance of newly generated components in the PE group suggests that the MSTC interfacial particle layer may reduce direct contact between ATVO and light, oxygen, or pro-oxidant factors in the aqueous phase, thereby inhibiting oxidation product formation and maintaining the relative compositional stability of the volatile oil.

### 3.4. Relationship Between PE Physicochemical Properties and Pharmacokinetic Behavior

The pharmacokinetic results suggested that the MSTC-stabilized PE exerted differential effects on the in vivo behavior of α-asarone and β-asarone. For α-asarone, the PE group showed increasing trends in AUC and C_max_, indicating that this system may improve its gastrointestinal dispersion, release, and absorption. This may be related to the dispersion of ATVO within emulsion droplets, the increased oil–water interfacial contact area, the protective effect of the interfacial particle layer on the active component, and the interaction between the positive charge of chitosan and the gastrointestinal mucosa [3,28,45]. These factors may jointly contribute to the increased systemic exposure of α-asarone. In contrast, the overall exposure of β-asarone changed only slightly, while T_1/2_ and MRT tended to decrease, suggesting that the emulsion system may affect the release, distribution, metabolism or elimination of β-asarone differently from α-asarone. Since α-asarone and β-asarone are structural isomers, differences in spatial configuration, lipophilicity, interfacial partitioning and metabolic susceptibility may lead to distinct pharmacokinetic behaviors within the same emulsion system. Similar carrier-mediated differential absorption phenomena have been reported in multi-component natural product delivery systems and are generally attributed to differences in physicochemical properties, interfacial partitioning behavior and structural evolution under gastrointestinal conditions [46,47].

Notably, relatively large inter-individual variability was observed for some pharmacokinetic parameters, particularly in the PE group. This variability, together with the limited sample size (*n* = 6), may have reduced the statistical power to detect between-group differences. Moreover, metabolite formation, tissue distribution, and excretion were not directly investigated in the present study. Therefore, the potential influence of the MSTC-stabilized PE on the in vivo behavior of α-asarone and β-asarone requires further confirmation through studies with larger sample sizes, as well as metabolite identification, tissue distribution, and excretion analyses.

### 3.5. Limitations and Future Perspectives

From the perspective of formulation design, MSTC has certain advantages as a prescription-derived particle stabilizer. STC is derived from traditional Chinese medicine materials, while chitosan is a biocompatible derivative of natural chitin and has been widely used in drug delivery and biomedical materials [48,49]. Compared with conventional surfactant-stabilized emulsions, MSTC-stabilized PE constructs an interfacial barrier using solid particles, thereby reducing the use of small-molecule surfactants and conforming to the concept of green formulation design. However, the positively charged surface of chitosan-modified particles may interact with cell membranes, mucosal surfaces, or biological macromolecules [50]. In addition, the proteinaceous and fibrous components of STC may involve potential immunological reactions or gastrointestinal irritation risks [51]. Therefore, although this study preliminarily suggests the in vivo application potential of this system, further evaluations of cytotoxicity, hemolysis, gastrointestinal irritation, repeated-dose toxicity, and long-term biocompatibility are still required. Notably, relatively large inter-individual variability was observed for some pharmacokinetic parameters, particularly in the PE group. This variability, together with the limited sample size (*n* = 6), may have reduced the statistical power to detect between-group differences. Moreover, metabolite formation, tissue distribution, and excretion were not directly investigated in the present study. Therefore, the potential influence of the MSTC-stabilized PE on the in vivo behavior of α-asarone and β-asarone requires further confirmation through studies with larger sample sizes, as well as metabolite identification, tissue distribution, and excretion analyses.

In summary, chitosan-mediated composite modification transformed STC particles into functional particles with interfacial stabilization capability. The MSTC-stabilized PE maintained emulsion stability through interfacial adsorption, particle-film barrier formation, steric hindrance and electrostatic repulsion, while reducing ATVO volatilization loss and light-oxidative degradation and modulating the in vivo behavior of asarone-type constituents to some extent. This strategy provides a promising and potentially translatable green stabilization approach for improving the quality of volatile-oil-containing traditional Chinese medicine preparations. Future studies should further focus on batch-to-batch consistency, long-term stability, metabolic transformation, tissue distribution, and systematic safety evaluation.

## 4. Materials and Methods

### 4.1. Materials and Equipment

*Acorus tatarinowii* Schott volatile oil (OEC2001) and *Saigae Tataricae Cornu* powder were supplied by Poli Aromatic Pharmaceutical Technology Co., Ltd. (Shanghai, China); sodium thiosulfate, anhydrous sodium carbonate, sodium chloride, absolute ethanol, and glacial acetic acid were purchased from Tianjin Tianli Chemical Reagent Co., Ltd. (Tianjin, China); chitosan and potassium bromide were obtained from Shanghai Macklin Biochemical Technology Co., Ltd. (Shanghai, China); reference standards for α-asarone (J12HB180373) and β-asarone (J22HB174800) were provided by Shanghai Yuanye Biotechnology Co., Ltd. (Shanghai, China); methylene blue was purchased from Shanghai Metatron Instrument Co., Ltd. (Shanghai, China); Nile Red was purchased from Csnpharm (Chicago, IL, USA); Nile Blue was purchased from Sigma-Aldrich (St. Louis, MO, USA); water was distilled water; and the other reagents were analytically pure.

### 4.2. Preparation of MSTC

Chitosan (4.00 g) was dissolved in 200 mL of 1% (*w*/*v*) glacial acetic acid solution. The chitosan solution was magnetically stirred at room temperature for 4 h, followed by storage at 4 °C overnight. Simultaneously, STC powder (2.00 g) was dispersed in 100 mL of distilled water, stirred under identical conditions, and stored at 4 °C overnight. The two suspensions were subsequently mixed at a volume ratio of 5:3 (*v*/*v*) and magnetically stirred for an additional 4 h at room temperature. The resulting mixture was dried at 60 °C overnight under atmospheric pressure, cooled to room temperature, and finely ground to obtain MSTC.

### 4.3. Methods for Characterization of MSTC

#### 4.3.1. Contact Angle

The surface wettability of STC powder and MSTC was evaluated using the sessile drop method with a JY-82 contact angle goniometer (Chengde Testing Machine Co., Ltd., Chengde, China). Before measurement, each powder sample was compressed into circular discs with a diameter of 20 mm and a thickness of 5 mm. The sample discs were placed horizontally on the contact angle measurement platform. Under room temperature conditions, 5 μL of deionized water was carefully deposited onto the sample surface using the instrument dosing system, and images were captured within 5 s after the droplet contacted the sample surface. The left and right contact angles of the droplet were calculated using Image J. Three independently prepared discs were measured for each sample, and the results were expressed as mean ± SD (*n* = 3).

#### 4.3.2. Scanning Electron Microscopy (SEM) and Energy Dispersive Spectroscopy (EDS)

The surface morphology and elemental composition of the samples were examined using a ZEISS Sigma 300 scanning electron microscope (Carl Zeiss AG, Oberkochen, Germany). Prior to imaging, a small portion of each sample was mounted on a conductive adhesive and coated with a thin layer of gold using a Quorum SC7620 sputter coater operated at 10 mA for 45 s. Morphological characterization was performed at an accelerating voltage of 3–5 kV, while EDS elemental mapping was conducted at 15 kV to ensure adequate excitation of characteristic X-rays. All analyses were carried out under high-vacuum conditions to ensure optimal image resolution and accurate elemental detection.

#### 4.3.3. Fourier Transform Infrared Spectrum (FTIR)

Appropriate amounts of *Saigae Tataricae Cornu* powder, chitosan, and their physical mixture (1:1, *w*/*w*), and MSTC were blended with dry potassium bromide at a 1:100 ratio and ground to a fine powder in a mortar. The resulting mixture was pressed into transparent pellets using a hydraulic press under a pressure of 8 N. FTIR measurements were carried out in the range of 4000–400 cm^−1^.

#### 4.3.4. Differential Scanning Calorimetry (DSC)

DSC was performed to characterize the thermal transitions of STC powder, chitosan, their 1:1 (*w*/*w*) physical blend, and MSTC. Each sample was precisely weighed and loaded into an aluminum pan, with an empty pan used as the reference. Analyses were conducted under a nitrogen purge. After equilibration at 25 °C, the temperature was ramped to 270 °C at 10 °C·min^−1^, while heat-flow signals were continuously recorded over the entire heating program.

### 4.4. Preparation of PE

For the preparation of the PE, 0.30 g of MSTC was added to a 50 mL centrifuge tube containing 10 mL of ATVO and 10 mL of water. The mixture was subjected to high-speed shearing at 14,000 rpm for 4 min to obtain a PE stabilized by 1.5% (*w*/*v*) MSTC.

### 4.5. Methods for Characterization of PE

#### 4.5.1. Type Determination

A suitable aliquot of the PE was collected and independently combined with an equal volume of 1% methylene blue aqueous solution or 0.5% Sudan III solution in 90% (*v*/*v*) ethanol. The mixtures were shaken thoroughly and allowed to stand for 10 min to ensure complete staining. Subsequently, a small portion of the stained emulsion was placed on a clean glass slide, and observations were performed under a microscope using a 40× objective lens. The dye distribution was used to determine the staining characteristics of the MSTC-stabilized PE.

#### 4.5.2. Size, PDI and Zeta Potential

The droplet size distribution, PDI, and zeta potential of the PE were determined using a Zetasizer Nano ZS90 dynamic light scattering (DLS) instrument (Malvern Instruments), Malvern, UK. Droplet size was expressed as D90 (nm). Three independently prepared discs were measured for each sample, and the results were expressed as mean ± SD (*n* = 3).

#### 4.5.3. Confocal Laser Scanning Microscopy (CLSM)

Fluorescence staining of the PE was performed following a modified procedure reported by Ru et al. [37]. Briefly, 1 mL of emulsion was combined with 100 µL of a mixed dye solution consisting of Nile Red (1 mg·mL^−1^ in isopropanol) and Nile Blue (1 mg·mL^−1^ in water). The mixture was vortexed for 5 min and incubated for 1 h to achieve complete staining. CLSM imaging was conducted with excitation at 488 nm (Nile Red) and 633 nm (Nile Blue). Nile Red labeled the oil phase (green fluorescence), while Nile Blue labeled the MSTC (red fluorescence). Fluorescence micrographs were collected for structural characterization.

#### 4.5.4. Storage Stability Evaluation of PE

Freshly prepared PE were stored at room temperature. The droplet size and PDI were measured daily for 7 days using a particle size analyzer, and each sample was measured in triplicate. All assays were performed in triplicate.

The CI was further determined to evaluate the long-term physical stability of the emulsion during storage. Briefly, the freshly prepared emulsion was transferred into a transparent glass tube and stored at room temperature without disturbance. The height of the separated layer and the total height of the emulsion were recorded at predetermined time points (*n* = 3). The CI was calculated according to Equation (1).

CI (%) = H_s_/H_t_ × 100% (1)

In the formula, H_s_ represents the height of the separated layer, and H_t_ represents the total height of the emulsion.

### 4.6. Effects of PE on the Accelerated Oxidative Stability of ATVO Under Light

#### 4.6.1. Preparation of Samples

Untreated ATVO, the physical mixture (10 mL ATVO, 10 mL distilled water, and 0.3 g MSTC mixed uniformly with a glass rod), and the MSTC-stabilized PE were each placed into flat weighing bottles (40 × 25 mm). The samples were stored in a light-stability chamber with a light intensity of 4500 ± 500 lx and a controlled temperature of 25 ± 0.5 °C. Aliquots were collected after 1, 3, and 5 days of light exposure. The physical mixture and PE groups were centrifuged (10,000 rpm, 10 min) to achieve phase separation prior to analysis. All assays were performed in triplicate, and the obtained samples were maintained at 4 °C until analyzed.

Volatile oil retention rate (%) = V_t_/V_0_ × 100% (2)

In the formula, V_t_ represents the volume of ATVO after light exposure for 1, 3, and 5 days, respectively (mL); V_0_ represents the initial volume of ATVO in each group (mL).

#### 4.6.2. Determination of Peroxide Value (POV) and Malondialdehyde (MDA)

For POV analysis, 500 μL of each sample was transferred to a 50 mL conical flask containing 10 mL of chloroform-acetic acid solution and 1 mL of saturated KI. After standing in the dark for 3 min, 30 mL of ultrapure water and 1 mL of 1% (*w*/*v*) starch indicator were added. The liberated iodine was titrated with 0.001 M Na_2_S_2_O_3_ until the blue coloration disappeared. A reagent blank was run concurrently, and POV was calculated using Equation (2) [34,35].

(3) $$\mathrm{POV}=\frac{(V-V_{0})\times c\times 1000}{2m}$$

In the formula, V and V_0_ are the titration volumes (mL) of Na_2_S_2_O_3_ for the sample and blank, c is the Na_2_S_2_O_3_ concentration (mol·L^−1^), and m is the sample mass (g).

For MDA determination, 500 μL of volatile oil was diluted to 10 mL with a trichloroacetic acid/acetic acid solution and sonicated at 40 °C for 10 min. After cooling, the mixture was filtered, and 5.00 mL of the filtrate was mixed with an equal volume of 0.02 M thiobarbituric acid (TBA) solution, followed by heating at 90 °C for 40 min. The reaction solution was subsequently cooled in the dark. Afterward, 5.00 mL of chloroform was added, and the mixture was left undisturbed for 1 h to allow phase separation. The absorbance of the upper phase was recorded at 532 nm. MDA content was quantified using a calibration curve established with 1,1,3,3-tetraethoxypropane as the standard. Each sample was analyzed in triplicate.

#### 4.6.3. Determination of ATVO Components

The volatile components of ATVO were determined by gas chromatography-mass spectrometry (GC-MS) according to a previously reported method [33]. Samples from each treatment group were prepared before analysis. Briefly, 100 μL of each volatile oil sample was transferred into a 10 mL volumetric flask. Then, 100 μL of n-docosane internal standard solution (10 mg·mL^−1^) was added. The mixture was diluted to volume with n-hexane. The solution was dried with anhydrous sodium sulfate. It was then vortex-mixed and filtered through a 0.22 μm organic membrane filter. The filtrate was used for GC-MS analysis.

GC-MS analysis was performed using an HP-5MS fused-silica capillary column (30 m × 0.25 mm, 0.25 μm). High-purity helium was used as the carrier gas. The flow rate was 1.0 mL·min^−1^. The injection volume was 1 μL. The split ratio was 10:1. The injector temperature was set at 230 °C. The oven temperature program was as follows. The initial temperature was 50 °C and held for 2 min. The temperature was increased to 140 °C at 15 °C·min^−1^ and held for 2 min. It was then increased to 144 °C at 0.4 °C·min^−1^ and held for 5 min. Finally, the temperature was increased to 250 °C at 10 °C·min^−1^ and held for 2 min. Mass spectrometry was performed in electron ionization mode at 70 eV. The ion source temperature was 230 °C. The quadrupole temperature was 150 °C. The solvent delay was 3 min. Full-scan data were collected over an *m*/*z* range of 35–500.

### 4.7. Pharmacokinetic Characteristics

The pharmacokinetics of α-asarone and β-asarone were evaluated in male SPF-grade SD rats following the protocol described in Zhu et al. [45]. The rats were randomly assigned to three groups (*n* = 6 per group): blank, ATVO, and PE. The animals were allowed to acclimatize for 1 week and were fasted for 12 h prior to dosing, with water provided ad libitum. Rats in the ATVO and PE groups were orally gavaged with ATVO at 752 mg/kg, whereas the blank group received the same volume of normal saline. Approximately 1 mL of blood was collected from the orbital vein at 5, 15, 30, 45, 60, 90, 120, 180, 240, 360, 480, and 720 min post-administration. Blood samples were collected into heparinized tubes and centrifuged at 4000 rpm for 15 min. The resulting plasma was separated and stored at −80 °C until further analysis. Plasma proteins were precipitated by mixing 150 μL plasma with 150 μL n-hexane, vortexing for 5 min, and centrifuging at 12,000 rpm for 10 min. The supernatant was used for chromatographic analysis. Mixed standard solutions of α-asarone and β-asarone were prepared by diluting with n-hexane. Calibration curves were constructed by spiking blank plasma with a series of standard concentrations and plotting peak area versus concentration. Quality control (QC) samples at low, medium, and high concentration levels were prepared to assess the precision, accuracy, extraction recovery, and matrix effect of the analytical method. The animal experiment was approved by Shaanxi University of Chinese Medicine’s Animal Ethics Committee, approval No.: SUCMDL20241227003.

### 4.8. Statistical Analysis

All data are presented as mean ± standard deviation (SD) from at least three independent experiments. Statistical analyses were performed using SPSS Statistics 26 (IBM Corporation, Armonk, NY, USA). Pharmacokinetic parameters were calculated using a non-compartmental model in DAS 2.0 software, including C_max_, T_max_, and AUC. Group comparisons were conducted using analysis of variance (ANOVA) or appropriate non-parametric tests. Statistical significance was defined as *p* < 0.05, with higher significance levels indicated by *p* < 0.01 or *p* < 0.001.

Compound annotation was performed by comparing the acquired EI mass spectra with the NIST 14.L mass spectral library. The database output, including retention time (RT), library-assigned compound name (Library/ID), CAS number, and match quality score (Qual), was used to evaluate the reliability of compound annotation. To reduce the influence of low-confidence library matches on subsequent data interpretation, compounds with Qual values lower than 60 were excluded during data integration and component screening. Relative quantification was performed using n-docosane as the internal standard. Untreated ATVO was used as the control sample. Differential components were screened using the limma package in R. Components with a |log_2_ fold change| ≥ 1.5 were considered significantly changed. Volcano plots, heatmaps, temporal trend plots, Venn diagrams, UpSet plots, and PCA plots were used to visualize the changes in volatile components among different groups.

Pharmacokinetic parameters were calculated using a non-compartmental model in DAS software. The calculated parameters included C_max_, T_max_, AUC, MRT, and T_1/2_. The pharmacokinetic parameters of α-asarone and β-asarone were analyzed separately.

## 5. Conclusions

In this study, STC powder was surface-engineered via chitosan-based composite modification to obtain stabilizing particles capable of efficient adsorption at the oil–water interface. Using these particles, an ATVO-loaded PE was successfully constructed, enabling interfacial-barrier encapsulation of ATVO. CLSM observations confirmed that ATVO was mainly distributed within the emulsion droplets and that a distinct particulate shell was formed at the interface; the droplets showed a uniform size distribution, indicating good physical stability of the system. Under light-exposure conditions, peroxide levels in the PE group were significantly lower than those in the free ATVO group at days 1, 3, and 5, suggesting effective suppression of oxidative progression. GC-MS combined with differential analysis and PCA further demonstrated that compositional divergence among groups increased with prolonged light; however, the PE showed smaller fluctuations and more effectively retarded relative changes in major constituents, including α-asarone, β-asarone, and γ-asarone. In vivo pharmacokinetic results revealed a differential modulation of asarone isomers by the emulsion system, with a pronounced increase in systemic exposure of α-asarone, whereas overall exposure of β-asarone changed only marginally. Collectively, these findings indicate that the MSTC particle-stabilized PE can simultaneously enhance the light-oxidative stability of ATVO and improve its in vivo exposure, providing a valuable and translatable strategy for stabilizing volatile oils and improving the quality of volatile-oil-containing TCM solid preparations.

## Figures and Tables

**Table 1:** Surface element distribution of STC, chitosan and MSTC.

Sample	Element	Line Type	Wt%	Wt% Sigma	At%
*Saigae Tataricae Cornu*	C	K	59.72	1.05	70.84
	N	K	12.89	1.19	13.11
	O	K	8.59	0.35	7.65
	Mg	K	0.01	0.07	0.01
	P	K	3.13	0.22	1.44
	S	K	15.60	0.39	6.93
	Ca	K	0.05	0.21	0.02
	Zn	L	0.00	0.14	0.00
chitosan	C	K	62.60	0.41	67.87
	N	K	16.27	0.52	15.13
	O	K	20.46	0.20	16.65
	Mg	K	0.66	0.03	0.35
MSTC	C	K	74.71	0.35	83.05
	O	K	15.45	0.26	12.89
	Mg	K	0.19	0.05	0.10
	P	M	1.71	0.11	0.74
	S	K	6.69	0.13	2.79
	Cl	K	0.61	0.08	0.23
	Ca	K	0.58	0.10	0.19
	Zn	L	0.06	0.10	0.01

**Table 2:** Volatile oil retention rate of different ATVO formulations after light exposure (*n* = 3, mean ± SD).

Time (d)	O	M	P
1	89.14 ± 0.61	91.75 ± 0.53 **	93.53 ± 0.29 ***
3	75.74 ± 0.65	79.60 ± 0.39 ***	81.85 ± 0.37 ***
5	64.38 ± 0.30	67.75 ± 0.62 **	71.56 ± 0.48 ***

Note: ** *p* < 0.01, *** *p* < 0.001 compared with the ATVO group treated with light for the same duration; O, ATVO group; M, mixture group; P, PE group.

**Table 3:** Peroxide values of different ATVO formulations after light exposure (*n* = 3, mean ± SD).

Time (d)	O	M	P
1	48.44 ± 0.19	42.15 ± 0.09 ***	37.06 ± 0.07 ***
3	70.04 ± 0.06	64.31 ± 0.04 ***	57.95 ± 0.07 ***
5	95.74 ± 0.04	80.01 ± 0.14 ***	68.61 ± 0.09 ***

Note: *** *p* < 0.001 compared with the ATVO group treated with light for the same duration; O, ATVO group; M, mixture group; P, PE group.

**Table 4:** Malondialdehyde contents of different ATVO formulations after light exposure (*n* = 3, mean ± SD).

Time (d)	O	M	P
1	0.38 ± 0.01	0.35 ± 0.03	0.28 ± 0.02 **
3	0.63 ± 0.01	0.53 ± 0.03 **	0.51 ± 0.01 ***
5	0.75 ± 0.02	0.65 ± 0.01 **	0.59 ± 0.03 **

Note: ** *p* < 0.01, *** *p* < 0.001 compared with the ATVO group treated with light for the same duration; O, ATVO group; M, mixture group; P, PE group.

**Table 5:** Pharmacokinetic parameters of α-asarone and β-asarone (*n* = 6, mean ± SD).

Pharmacokinetic Parameters	α-Asarone		β-Asarone	
	ATVO	PE	ATVO	PE
AUC_0–t_ (mg/L·h)	72.47 ± 33.89	146.67 ± 128.07	160.59 ± 69.89	164.00 ± 59.32
AUC_0–∞_(mg/L·h)	149.99 ± 101.70	594.26 ± 912.79	255.88 ± 82.78	280.67 ± 279.36
MRT_0–t_ (h)	6.17 ± 0.40	5.84 ± 0.71	5.88 ± 0.48	5.45 ± 0.59
MRT_0–∞_ (h)	9.75 ± 4.45	19.32 ± 27.47	13.59 ± 7.83	7.22 ± 2.78
T_1/2_ (h)	5.15 ± 2.02	11.95 ± 18.25	7.86 ± 6.43	3.87 ± 1.85
T_max_ (h)	4.81 ± 5.17	3.51 ± 3.62	4.88 ± 4.61	4.42 ± 3.01
C_max_ (mg/L)	9.91 ± 4.90	24.51 ± 21.13	21.22 ± 10.11	23.93 ± 8.70

## Data Availability

The original contributions presented in this study are included in the article/Supplementary Materials. Further inquiries can be directed to the corresponding author(s).

## Supplementary Materials

The following supporting information can be downloaded at: https://www.mdpi.com/article/10.3390/ph19071027/s1, Figure S1: Chromatogram of plasma sample; Table S1: Standard curves of β-asarone and α-asarone; Table S2: Intra-day and inter-day precision of β-asarone and α-asarone in rat plasma; Table S3: Extraction recovery and matrix effects of β-asarone and α-asarone in rat plasma; Table S4: Stability of β-asarone and α-asarone in rat plasma; Table S5: GC-MS identification information of volatile components detected in ATVO under light exposure.
