# Supplementary material for: Pickering Emulsion Stabilized by Chitosan-Modified Saigae Tataricae Cornu Particles for Improving the Oxidative Stability and In Vivo Pharmacokinetics of Acorus tatarinowii Schott Volatile Oil
Source: Pharmaceuticals (Basel). 2026 Jun 30;19(7):1027. doi: 10.3390/ph19071027 (PMC13415033; doi:10.3390/ph19071027)
Supplement: Supplementary file 1 [file pharmaceuticals-19-01027-s001.zip › pharmaceuticals-4329142-supplementary.pdf]

Supplementary Material

1. *In vivo* pharmacokinetic studies

1.1 Specificity

Fig. S1 shows that at the retention time of β-asarone and α-asarone, there was no interference from the blank solvent, indicating good specificity of this method.

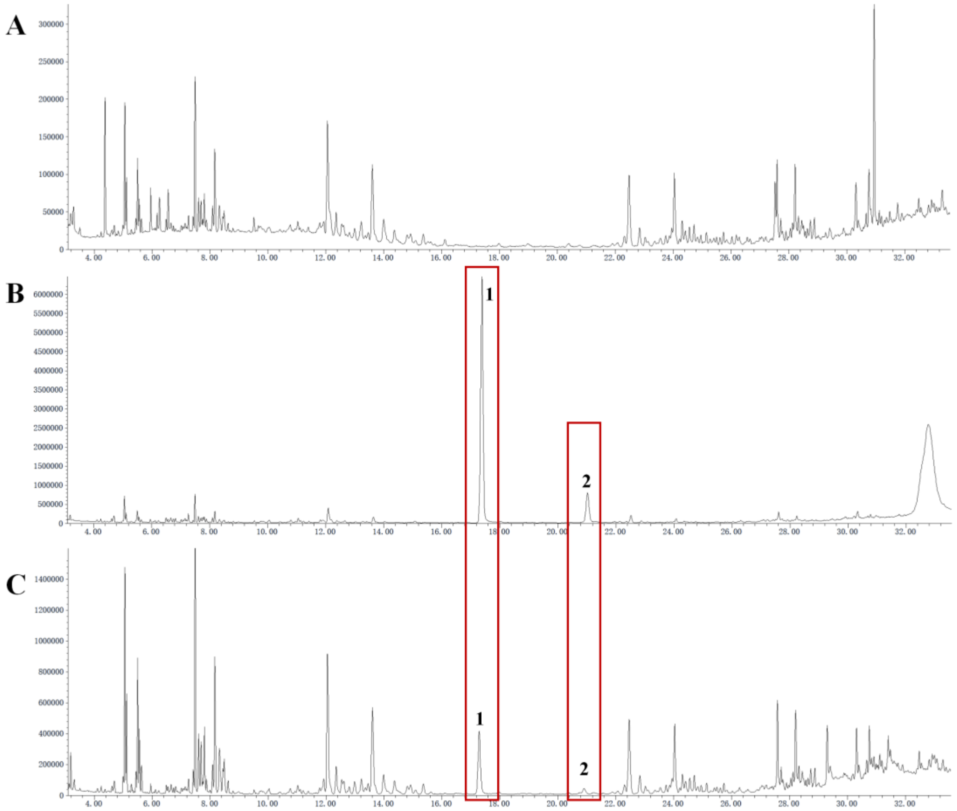

Figure S1 Chromatogram of plasma sample. A blank plasma; B blank plasma spiked with control samples; C plasma after administration. 1 β-asarone; 2 α-asarone

1.2 Standard curve

Aliquots of blank plasma (150 μL) were accurately transferred and spiked with a series of mixed working standard solutions containing β-asarone and α-asarone. The resulting plasma concentrations were 147, 73.5, 36.75, 18.375, 7.35, and 3.675 μg/mL for β-asarone, and 26.068, 13.034, 6.517, 3.2585, 1.3034, and 0.6517 μg/mL for α-asarone. Calibration curves were constructed by plotting the concentrations of

α-asarone and β-asarone against their corresponding peak areas, and the regression equations and linear ranges were calculated. As shown in Table S1, the correlation coefficients (R^2) for both analytes were greater than 0.99, indicating good linearity for β-asarone and α-asarone within their respective concentration ranges.

Table S1 Standard curves of β-asarone and α-asarone

Components	Regression equation	R^2	Linear range（μg/mL）
β-asarone	Y = 1399586.7883 X - 4856661.8127	0.9992	3.675 - 147
α-asarone	Y = 1017947.3604 X - 909957.9991	0.9986	0.6517 - 26.068

1.3 Precision

Plasma quality control (QC) samples of β-asarone and α-asarone were prepared at low, medium, and high concentration levels. Six replicates of each concentration were analyzed continuously within 24 h to determine intra-day precision, and the assay was repeated over 3 consecutive days to evaluate inter-day precision. As shown in Table S2, the intra-day and inter-day precision values (RSD) for both β-asarone and α-asarone at all three concentration levels met the methodological requirements.

Table S2 Intra-day and inter-day precision of β-asarone and α-asarone in rat plasma (n = 6).

Components	Concentration（μg/mL）	Intra-day RSD (%)	Inter-day RSD (%)
β-asarone	102.90	3.81	6.37
	29.40	5.05	11.72
	5.88	10.12	9.44
α-asarone	18.2476	5.10	8.48
	5.2136	4.73	13.18

	1.04272	10.98	14.89

1.4 Extraction recovery and matrix effects

Plasma QC samples of β-asarone and α-asarone at low, medium, and high concentration levels were prepared and analyzed according to the plasma sample pretreatment procedure, with six replicates for each concentration. The resulting peak areas were recorded as A. Blank plasma samples were processed in the same manner and then spiked with the corresponding QC samples at low, medium, and high concentrations, with six replicates for each level; the measured peak areas were recorded as B. Standard solutions of β-asarone and α-asarone at low, medium, and high concentrations were diluted with n-hexane and analyzed directly, and the corresponding peak areas were recorded as C. Extraction recovery was calculated as A/B × 100%, and matrix effect was calculated as B/C × 100%. As shown in Table S3, the extraction recoveries and matrix effects of both β-asarone and α-asarone ranged from 85.00% to 115.00%, meeting the acceptance criteria.

Table S3 Extraction recovery and matrix effects of β-asarone and α-asarone in rat plasma (n = 6).

Components	Concentration (μg/mL)	Extraction recovery (%)	RSD (%)	Matrix effects (%)	RSD (%)
	102.90	92.90	3.61	102.89	9.41
β-asarone	29.40	94.74	4.55	103.57	5.38
	5.88	103.52	3.81	107.58	5.94
	18.2476	95.50	4.50	107.73	4.73
α-asarone	5.2136	94.74	4.55	103.57	5.38
	1.04272	100.93	6.29	100.47	10.46

1.5 Stability

QC samples at low, medium, and high concentrations were subjected to room temperature stability (24 hours at room temperature) and freeze-thaw stability (three cycles of freeze-thaw). Six replicates were prepared for each concentration, and the results are presented in Table S4. Overall, both analytes exhibited relatively small stability fluctuations across the different concentration levels. Although β-asarone and α-asarone at the medium concentration level showed comparatively greater variation under freeze–thaw conditions, the results remained within an acceptable range and were considered suitable for subsequent quantitative analysis of plasma samples.

Table S4 Stability of β-asarone and α-asarone in rat plasma（n = 6）

Components	Concentration (μg/mL)	Short-term stability RSD (%)	Freeze-thaw stability RSD (%)
	102.90	2.43	8.93
β-asarone	29.40	8.51	11.31
	5.88	8.92	5.92
	18.2476	1.62	9.13
α-asarone	5.2136	10.52	12.90
	1.04272	10.76	10.10

Table S5 GC-MS identification information of volatile components detected in ATVO under light exposure

CAS	Library/ID	Ref	Qual	RT
003779-61-1	1,3,6-Octatriene, 3,7-dimethyl-, (E)-	40369	98	4.9602
000078-70-6	Linalool	71793	97	5.5107
000629-62-9	Pentadecane	231834	93	7.488
000515-13-9	1-Methyl-1-ethenyl-2,4-bis(1'-methylethenyl)cyclohexane	204393	99	9.6035
006831-16-9	(-)-Aristolene	204001	99	10.3193
017334-55-3	1H-Cyclopropa[a]naphthalene, 1a,2,3,5,6,7,7a,7b-octahydro-1,1,7,7a-tetramethyl-, [1aR-(1a.alpha.,7.alpha.,7a.alpha.,7b.alpha.)]-	204285	98	10.6404
006380-24-1	(Z)-Methyl isoeugenol	127050	98	11.6184
021747-46-6	1H-Cycloprop[e]azulene, 1a,2,3,5,6,7,7a,7b-octahydro-1,1,4,7-tetramethyl-, [1aR-(1a.alpha.,7.alpha.,7a.beta.,7b.alpha.)]-	204700	98	12.37
039020-72-9	(2R,3R,6S)-6-Isopropyl-3-methyl-2-(prop-1-en-2-yl)-3-vinylcyclohexanone	257596	99	12.9149
000483-76-1	.delta.-Cadinene	204022	97	13.2406
021698-46-4	(3S,6S)-6-Isopropyl-3-methyl-2-(propan-2-ylidene)-3-vinylcyclohexanone	258321	99	13.4987
021391-99-1	.alpha.-Calacorene	192081	97	14.03
000487-11-6	Benzene, 1,2,3-trimethoxy-5-(2-propenyl)-	216204	98	14.306
005353-15-1	.gamma.-Asarone	216178	97	15.0936
086000-71-7	Naphthalene, 1,2,3,4,5,8-hexahydro-6-methoxy-7-methyl-1-(1-methylethyl)-, (.+-.)-	257398	86	16.7209
002883-98-9	Asarone	216643	98	17.9034
1005276-30-1	Dehydroxy-isocalamendiol	257843	99	18.1124
997276-05-6	8-Aristolen-10.alpha.-ol	257938	90	19.8309
005273-86-9	.beta.-Asarone	216643	98	21.4921
004460-86-0	Benzaldehyde, 2,4,5-trimethoxy-	177502	99	22.9709
997340-11-2	Isocalamendiol	320570	91	23.8061

002020-90-8	1-(2,4,5-Trimethoxyphenyl)propan-2-one	270700	91	23.9747
000629-97-0	Docosane	571714	99	30.9817
003856-25-5	Copaene	204642	99	9.315
021698-44-2	(2S,3S,6S)-6-Isopropyl-3-methyl-2-(prop-1-en-2-yl)-3-vinylcyclohexanone	257597	99	12.9322
128487-46-7	Spirojatamol	265298	96	19.4486
003260-45-5	2,5-bis(1H-pyrrol-2-yl)-1H-pyrrole	181902	78	27.716
000000-00-0	6H-[2]benzopyrano[4,5-c]pyridin-6-one	181816	78	27.7146
000638-67-5	Tricosane	616619	99	31.9549
000119-47-1	Phenol, 2,2'-methylenebis[6-(1,1-dimethylethyl)-4-methyl-	665250	99	33.4098
997883-77-5	(+-)-Magnosalicin	855055	83	31.6291
019912-62-0	4-Isopropyl-1,6-dimethyl-1,2,3,4,4a,7,8,8a-octahydro-1-naphthalenol	265715	95	19.4505
997343-55-0	2-Benzyl-4-methylbenzo[d]thiazole	323949	64	27.5028
997883-77-6	(2R,3R,4S,5S)-2,4-diasaryl-3,5-dimethyl-tetrahydrofuran	855056	83	31.6243
000620-02-0	5-Methyl furfural	13353	93	4.1701
073006-77-6	[1,1'-Biphenyl]-2-amine, 4,5-dimethyl-	182390	78	27.8033
000629-94-7	Heneicosane	524705	98	29.4951
000629-92-5	Nonadecane	427520	98	31.952
105689-15-4	Acetamide, N-(3-phenyl-4-isothiazolyl)-	247691	64	25.0467
997499-20-0	1-Hydroxy-2-butanone oxime, o-[(pentafluorophenyl)methyl]-	476768	64	28.2281
110983-42-1	2-((E)-2-[4-(Dimethylamino)phenyl]ethenyl)phenol	324338	64	27.5028
041346-83-2	Formamide, N-(3,3-diphenylpropyl)-	324219	60	27.5027
000096-76-4	2,4-Di-tert-butylphenol	210958	97	12.7042
000112-95-8	Eicosane	476359	95	31.959
997895-04-0	2',4',6'-Trinitro-5'-phenyl-1,1' : 3',1-terphenyl"	866149	72	31.6308
997895-49-5	2-amino-5-(4-methoxyphenyl)-4-phenyl-4-(4-phenylphenyl)-3-pyrrolecarbonitrile	866598	90	31.9486
000135-77-3	1,2,4-Trimethoxybenzene	101356	97	9.0252

000110-27-0	Isopropyl myristate	434095	99	25.8654
000057-10-3	n-Hexadecanoic acid	384339	68	28.223
000593-45-3	Octadecane	377947	78	31.9486
001330-86-5	Diisooctyl adipate	742688	90	33.0263
077573-53-6	1(2H)-Naphthalenone, 6-acetyloctahydro-8a-methyl-, (4a.alpha.,7.beta.,8a.beta.)-	217560	60	24.0887
500350-54-9	5H-Pyrrolo[3,4-b]pyrazine-5,7(6H)-dione, 6-(phenylmethyl)-	323187	91	27.5028
007216-56-0	2,4,6-Octatriene, 2,6-dimethyl-, (E,Z)-	40305	97	5.9369
997342-50-3	.alpha.-Tetraloxime, 8-fluoro-5,6-dimethoxy-	322916	64	27.482
000545-47-1	Lupeol	847271	98	32.3147
006831-17-0	2H-Cyclopropa[a]naphthalen-2-one, 1,1a,4,5,6,7,7a,7b-octahydro-1,1,7,7a-tetramethyl-, (1a.alpha.,7.alpha.,7a.alpha.,7b.alpha.)-	250745	95	31.9278

Note: Library-matched compound name (Library/ID); library reference number (Ref); match quality score (Qual); retention time (RT).
